# Monte Carlo Analysis of Optical Interactions in Reflectance and Transmittance Finger Photoplethysmography

**DOI:** 10.3390/s19040789

**Published:** 2019-02-15

**Authors:** Subhasri Chatterjee, Panayiotis A. Kyriacou

**Affiliations:** Research Centre for Biomedical Engineering (RCBE), City, University of London, London EC1V 0HB, UK; p.kyriacou@city.ac.uk

**Keywords:** photoplethysmography, calibration curve, pulsatile tissue, oxygen saturation, Monte Carlo, scattering and absorption

## Abstract

Photoplethysmography (PPG) is a non-invasive photometric technique that measures the volume changes in arterial blood. Recent studies have reported limitations in developing and optimising PPG-based sensing technologies due to unavailability of the fundamental information such as PPG-pathlength and penetration depth in a certain region of interest (ROI) in the human body. In this paper, a robust computational model of a dual wavelength PPG system was developed using Monte Carlo technique. A three-dimensional heterogeneous volume of a specific ROI (i.e., human finger) was exposed at the red (660 nm) and infrared (940 nm) wavelengths in the reflectance and transmittance modalities of PPG. The optical interactions with the individual pulsatile and non-pulsatile tissue-components were demonstrated and the optical parameters (e.g., pathlength, penetration depth, absorbance, reflectance and transmittance) were investigated. Results optimised the source-detector separation for a reflectance finger-PPG sensor. The analysis with the recorded absorbance, reflectance and transmittance confirmed the maximum and minimum impact of the dermis and bone tissue-layers, respectively, in the formation of a PPG signal. The results presented in the paper provide the necessary information to develop PPG-based transcutaneous sensors and to understand the origin of the ac and dc components of the PPG signal.

## 1. Introduction

Photoplethysmography (PPG) is a non-invasive technique that uses light for measuring the volumetric changes in blood associated with the cardiac cycle in vascular tissue beds [1]. In PPG, a volume of peripheral tissue is illuminated by an optical radiation that undergoes multiple events of scattering and absorption as it traverses through different tissue-layers and finally is transmitted through or reflected from the tissue volume. The modulation of the light absorbance in the tissue due to the fluctuations in blood volume between systole and diastole gives rise to the PPG waveform [2]. A PPG waveform is divided into two components: the pulsatile component (ac) which varies in synchrony with the cardiac cycle, and the slowly varying component (dc) [3]. A PPG waveform is most often acquired by the system named Pulse Oximetry, which is the standard procedure for the clinical measurement and monitoring of arterial blood oxygen saturation $\left(SpO_{2}\right)$ [4]. The arterial oxygen saturation is derived from the relative change in amplitudes of red and near-infrared light by the main two absorbers present in blood, namely, oxyhaemoglobin ($HbO_{2}$) and deoxyhaemoglobin (HHb) [2]. Transmittance mode finger pulse oximeters are the most common application of PPG, whereas the reflectance mode PPG probes are more flexible regarding its location, and suitable as wearable sensors.

In recent years, there has been a plethora of interest in extending the application of PPG beyond pulse oximetry, for example, usage of the PPG in the assessment of vascular mechanics, blood pressure, blood viscosity, pulse transit time estimation, pulse rate variability, assessment of tissue perfusion etc. [1,3,5,6,7,8,9,10]. With the growing interest in PPG research, it is imperative to have a precise understanding of the fundamental aspects of light-tissue interactions in PPG. Recent studies [3,11,12] have revealed the importance of the knowledge about optical pathlength and penetration depth in PPG. Such parameters depend on the operating optical wavelength, sensor geometry and the ‘region of interest’ (ROI). Different ROIs in body exhibit different anatomical configurations with multi-scale heterogeneity and structural complexity [13]. For an adequate understanding, therefore, an in-depth analysis of optical interactions with a specific ROI pertinent to the PPG application is crucial.

Previous mathematical models of PPG and pulse oximetry based on Beer-Lambert law, simple diffusion theory or random walk theory [14,15,16] were inadequate to analyse the light-tissue interactions in a highly scattering and absorbing tissue medium. Later on, Monte Carlo method-based heterogeneous tissue models including vascular distribution were implemented for various applications in PPG by different research groups [17,18,19,20,21]. However, all the available models were concerned with the effect of cutaneous vasculature in the reflectance modality of PPG only. The optical interactions with all absorbers (i.e., blood, water and melanin) present in all pulsatile and non-pulsatile compartments (including skin, fat, bone and muscle) of a tissue volume has never been investigated. In other words, no research work is yet available to demonstrate the optical interactions with all tissue layers in a specific ROI in both reflectance and transmittance modalities of PPG.

In this paper, a light-tissue interaction-based model of a dual wavelength PPG sensor is presented where a heterogeneous tissue-volume resembling human finger has been exposed at the commonly used optical wavelengths: red (660 nm) and infrared (940 nm). Monte Carlo method has been chosen for the analysis which is a stochastic approach and is extensively used for simulating light propagation through biological tissues [22]. The method provides several important advantages over other approaches (e.g., diffusion approximation, random walk model etc.) which include [23]—(a) the flexibility regarding the size, shape and position of the optical source and detector; (b) inclusion of any level of complexity and heterogeneity in the tissue structure; (c) incorporating all physical processes between light and tissue such as multiple scattering, scattering anisotropy, high absorption, reflection and refraction etc.; (d) ability to produce accurate results. The model presented in this paper has been executed to asses the contribution of different tissue layers in the formation of the ac and dc components of a finger-PPG signal.

## 2. Methodology

### 2.1. An Anatomical Feature of the Tissue Model

**Figure 1:**
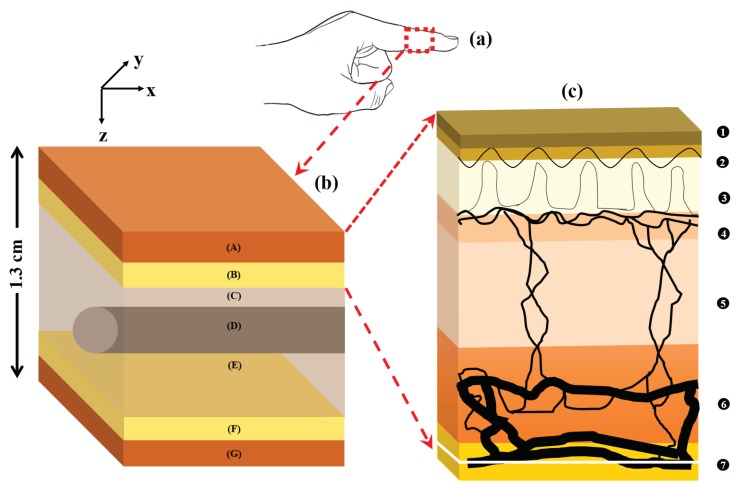
A volume of the finger tissue (**a**) is zoomed in (**b**) where the tissue layers are described as: skin (A), fat (B) and muscle (C,E), and then fat (F) and skin (G) in the reverse order. The muscle layer also contains a cylindrical bone (D) within it. The vasculature in the skin tissue sublayers (1–7) is illustrated in (**c**) and the stratification are described in Table 1.

The anatomical feature of the tissue model is presented in Figure 1. The overall geometry of the volume of the index finger, shown in Figure 1a, was presented by a three-dimensional semi-infinite slab. As shown in Figure 1b, the heterogeneous volume of the index finger had a thickness of 1.3 cm and contained the following layers [24,25,26,27]: (A) skin sublayers, (B) fat, (C & E) muscle, and then (F) fat and (G) skin sublayers in the reverse order. The muscle layer contained a cylindrical bone (D) of a diameter of 4 mm at a depth of 5 mm from the top surface [13,28]. The muscle layer was considered 10 mm thick and represented the overall fibrous tissue-network surrounding the bone such as tendons and ligaments (e.g., annular pulleys and cruciate pulleys) that are attached to the lumbrical muscle [27,29,30]. The skin layer had a total thickness of 0.95 mm [13,31] and comprised six sublayers [23,26,32,33]: (1) stratum corneum; (2) epidermis; (3) papillary dermis; (4) upper blood net dermis; (5) reticular dermis and (6) deep blood net dermis. In Figure 1c, the vascular distribution in the skin sublayers (1-6), followed by the subcutaneous fat layer (7), is illustrated.

The ‘pulse’ was simulated in the tissue by increasing the arterial blood volume in the dermis during systole by twice as much as in diastole [24]. The systolic increase in pulsatile tissue volume was associated with an equivalent decrease in the volume of non-pulsatile tissue compartments. The ratio of arterial and venous dermal blood was 1:1 [34]. Venous oxygen saturation was considered 10% lower than the arterial oxygen saturation [35]. An epidermal melanin concentration of 10% was considered in the model [36]. The effect of skin hydration was taken into consideration. Parameters used to simulate the dermal sublayers, i.e., the thickness (t), the baseline (i.e., diastolic) blood volume $\left(V_{b}\right)$ and the volume of water in the dermal sublayers $\left(V_{w}\right)$ are illustrated in Table 1.

### 2.2. Tissue Optical Properties

The optical parameter responsible for attenuating light propagation through tissue is its absorption coefficient $\mu_{a}$. The baseline absorption coefficient $\mu_{a_{baseline}}$ (i.e., the absorption coefficient of the dermal sublayer due to its intrinsic absorption property only in absence of any other chromophore) at an operating wavelength λ is expressed by the equation below [38,39]:

(1) $$\mu_{a_{baseline}}\left(\lambda \right)=7.84\times 10^{7}\times \lambda^{-3.255}.$$

Considering the absorbance of light through arterial and venous blood with different concentrations of the absorbers oxyhaemoglobin ($HbO_{2}$) and deoxyhaemoglobin (HHb), the total absorption coefficient of any i-th dermal sublayer can be written as: [14,23,32]

(2) $$\mu_{a_{i}}\left(\lambda \right)=V_{A_{i}}\mu_{a_{A_{i}}}\left(\lambda \right)+V_{V_{i}}\mu_{a_{V_{i}}}\left(\lambda \right)+V_{w_{i}}\mu_{a_{w_{i}}}\left(\lambda \right)+[1-\left(V_{A_{i}}+V_{V_{i}}+V_{w_{i}})\right]\mu_{a_{baseline_{i}}}\left(\lambda \right)$$

where $V_{A}$ and $V_{V}$ stand for the arterial and venous blood volume-fraction respectively. $\mu_{a_{A}}$, $\mu_{a_{V}}$ and $\mu_{a_{w}}$ are the absorption coefficients of the arterial blood, venous blood and water. Oxygen saturation, by definition, is the concentration of oxygen saturated haemoglobin in the total blood. Considering $SaO_{2}$ and $SvO_{2}$ are respectively the arterial and venous oxygen saturation, absorption coefficients of the arterial and venous blood can be written as:

(3) $$\begin{array}{c} \mu_{a_{A}}\left(\lambda \right)=SaO_{2}\mu_{a_{HbO_{2}}}\left(\lambda \right)+\left(1-SaO_{2}\right)\mu_{a_{HHb}}\left(\lambda \right)\\ \mu_{a_{V}}\left(\lambda \right)=SvO_{2}\mu_{a_{HbO_{2}}}\left(\lambda \right)+\left(1-SvO_{2}\right)\mu_{a_{HHb}}\left(\lambda \right) \end{array}$$

where $\mu_{a_{HbO_{2}}}$ and $\mu_{a_{HHb}}$ are the absorption coefficients of oxy and deoxyhaemoglobin, respectively.

The epidermal layer of the skin does not contain any amount of blood but only the absorbers melanin and water. The melanin absorption coefficient $\mu_{a_{mel}}$ is determined from the following equation [36,40]:

(4) $$\begin{array}{c} \mu_{a_{mel}}\left(\lambda \right)=6.6\times 10^{10}\times \lambda^{-3.33} \end{array}$$

which is used to derive the absorption coefficient for epidermis:

(5) $$\mu_{a_{epi}}\left(\lambda \right)=V_{mel}\mu_{a_{mel}}\left(\lambda \right)+V_{w_{epi}}\mu_{a_{w}}\left(\lambda \right)+[1-\left(V_{mel}+V_{w_{epi}})\right]\mu_{a_{baseline}}\left(\lambda \right).$$

The absorption coefficients of water, oxyhaemoglobin and deoxyhaemoglobin (at a hematocrit of 45%) were adapted from literature [41,42,43,44]. The absorption coefficients of subdermal fat and muscle were adapted from the published data measured from human skin ex vivo [45]. Due to lack of data on the optical properties of a finger bone, the optical properties of skull bone was used for the simulation [46]. The scattering coefficient and anisotropy factor of skin, muscle and bone were adapted from published studies [45,46]. The optical properties used in the simulation are stated in Table 2.

Since the dermis is responsible for the pulsatile volumetric changes in the PPG signal, and also each dermal sublayer is composed of different chromophore distribution, the contribution of the individual components in the absorption was considered, as already expressed by Equations (2)–(5). However, due to unavailability of precise vascular distribution for muscle and fat tissue, and also for the fact that those layers are much thicker than skin, the absorption coefficient of these layers were considered as bulk. The finger bone (phalanx) is supposed to be different anatomically from cranial bone, especially in the blood content, which eventually would create differences in their optical properties. However, the main concern in the present study is the relative absorbance of light in red and infrared wavelength and not the absolute absorbance, which allowed the compromise of the bone optical properties.

### 2.3. Monte Carlo Simulation

**Figure 2:**
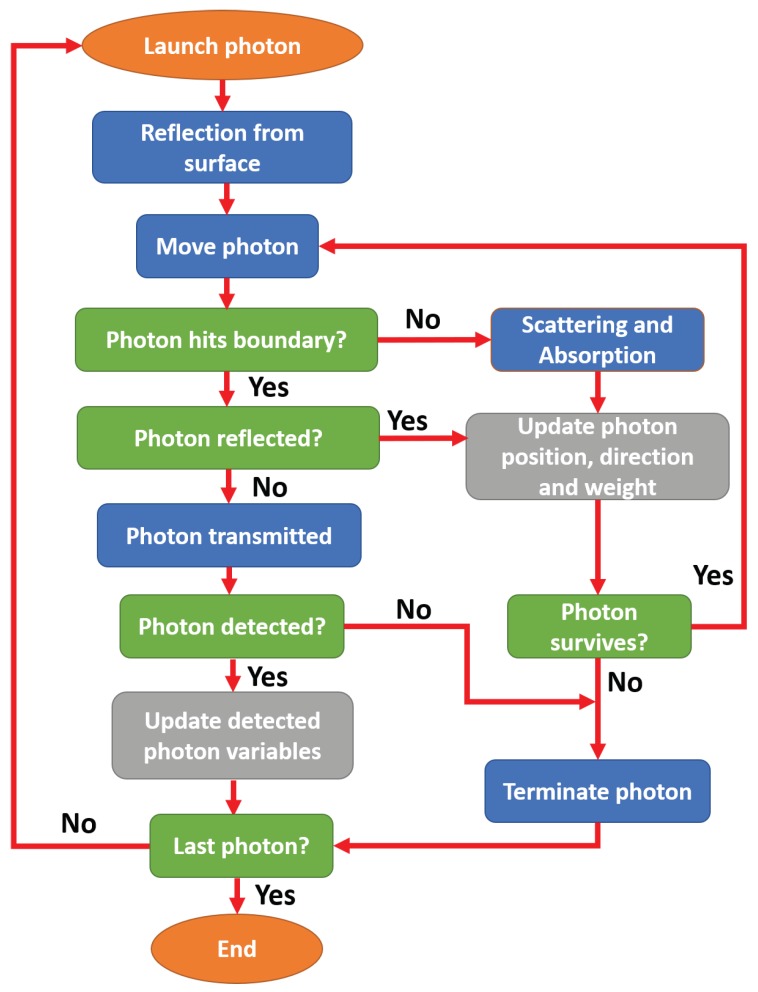
Flowchart of Monte Carlo algorithm.

A flowchart for the basic steps of the Monte Carlo (MC) simulation for propagation of light through tissue in a finger-PPG configuration is presented in Figure 2.

As shown, a photon packet with an initial direction and position co-ordinate was launched onto the tissue surface. The initial statistical weight of the photon packet was w=1. After the initial correction for the reflection at the tissue surface, the photon packet was propagated through a step-size (l), calculated by random sampling of the probability of photon scatter [47], i.e.,

(6) $$l=-\frac{ln(\xi )}{\mu_{s}}$$

where ξ is a computer-generated pseudo-random number (0<ξ<l). If the photon packet hit the boundary, a correction was made deciding whether it would reflect internally or transmit. If the photon transmitted, it was checked whether it had fallen within the detection criteria. If the photon was detected, several variables were scored (namely, optical path, detected intensity, and penetration depth), and the propagation of that photon packet was terminated. If the photon propagated freely, absorption and scattering events occurred. A certain fraction of the photon-weight (i.e., $\Delta w=\frac{\mu_{a}}{\mu_{a}+\mu_{s}}\cdot w$) was absorbed in the medium, and the rest of the weight continued to propagate. For the scattering, the direction of the photon packet was oriented through the randomly generated deflection and azimuthal angles. The scattering angle θ was calculated using the Henyey-Greenstein phase function [48] whereas the azimuth was randomly generated between 0 and 2π:

(7) $$\begin{array}{c} \theta =\cos^{-1}\frac{1}{2g}\left[1+g^{2}-{\left(\frac{1-g^{2}}{1-g+2g\xi }\right)}^{2}\right]\\ \;\phi =2\pi \xi . \end{array}$$

The same steps were repeated until the photon packet was detected or discarded. The photon was discarded if the photon weight was too small or it had transmitted without being detected, and a new photon packet was launched. The process would be repeated until a desired number of photon packets were detected.

A 64-bit Operating System with an installed memory of 24 GB and an Intel Xeon CPU (2.40 GHz, 2 processors) was dedicated for the simulation. A MATLAB (Mathworks, Inc., Natick, MA, USA) platform was chosen for coding and a multi-thread programming environment was used for facilitating the MC simulation.

### 2.4. Execution of the Model

As stated before, the MC model was executed in two modalities. In the reflectance mode, the optical source and detector were placed 5 mm apart whereas in the transmittance mode, the those were placed at two opposite surfaces of the tissue site (i.e., 13 mm apart). Incidence of a Gaussian beam of 1 mm radius was simulated to the tissue surface that propagated through the tissue to be detected at a circular detector of radius 1 mm. The MC model was executed to quantify the distribution of scattering events, and to calculate the mean optical path and penetration depth at red and infrared wavelengths. The model was also employed to record the ‘intensity’ of the detected photon packets. Here, ‘intensity’ *I* refers to the mean weight of the detected photon packets, which is termed as ‘reflectance’ or ‘transmittance’ at respective geometries. Expressing the ‘pulse’ (i.e., the difference between the diastolic and systolic intensity) by ΔI, the normalised pulsatile intensity $I_{N}$ was presented as:

(8) $$I_{N}\left(\lambda \right)=\frac{\Delta I(\lambda )}{I_{diastolic}\left(\lambda \right)}.$$

The ratio of the red and infrared relative normalised intensities was quantified by the ‘ratio of ratios’ (*R*) as:

(9) $$R=\frac{I_{N}\left(red\right)}{I_{N}\left(IR\right)}$$

The model was executed to investigate the relationship among the penetration depth, optical path and source-detector separation in a reflectance geometry. The mean penetration depth was calculated as the mean of the highest penetration of each photon packet within the tissue. The mean optical path was calculated as the mean of the total simulated pathlength of the photon packets from the source to the detector.

To assess the contribution of different tissue layers in PPG, the relative absorbance was also calculated. Similar to Equations (8) and (9), the normalised absorbances *A* at red and infrared wavelengths (i.e., $A\left(\lambda \right)=\Delta A\left(\lambda \right)/A_{diastolic}\left(\lambda \right)$), were used to quantify the absorbance modulation ratio of $R_{M}$ [49]:

(10) $$R_{M}=\frac{A(red)}{A(IR)}.$$

## 3. Results

**Figure 3:**
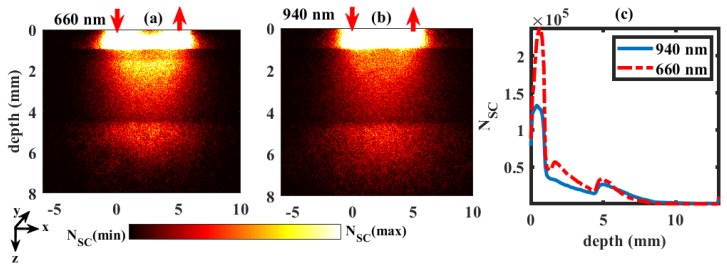
Scattering distributions at wavelengths 660 nm and 940 nm in the reflectance geometry are shown in (**a**,**b**), respectively. The upward and downward red arrows represent the position of optical source and detector. Colourbar represents the distribution between the maximum and minimum number of scattering events $\left(N_{SC}\right)$. The numbers of scattering at different depths of tissue are shown in (**c**).

The distribution of the scattering events in a dual wavelength reflectance PPG setting (similar to reflectance pulse oximetry) simulated by MC is shown in Figure 3. In this figure, a typical example of distribution through the tissue volume at $SaO_{2}$ = 90% is presented. The number of scattering events ($N_{SC}$) along the depth within the tissue volume for red and infrared wavelengths were shown in Figure 3a,b, respectively. The maximum number of scattering was found in the upper layers of the tissue volume, i.e., the dermal sublayers. No photon passed beyond the muscle layer in both red and infrared wavelengths (i.e., depth > 11.5 mm). The distributions of photon scatter with the penetration depth at both wavelengths are plotted in Figure 3c. The number of scattering events were higher in red compared to infrared wavelengths at smaller penetration depths. The deeper the light penetrated, the lesser the number of scattering events.

**Figure 4:**
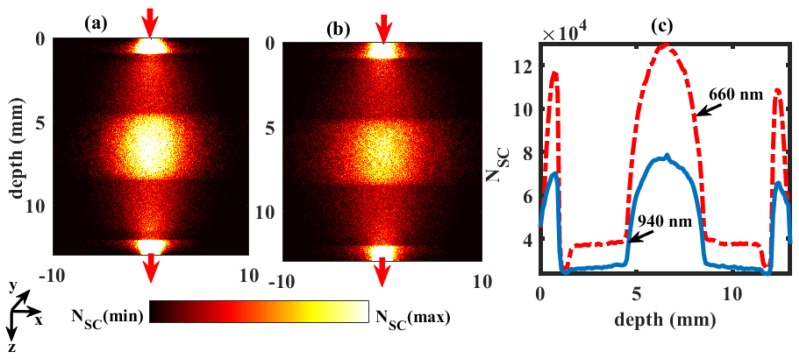
Scattering distributions at wavelengths 660 nm and 940 nm in the transmittance geometry are shown in (**a**,**b**), respectively. The upward and downward red arrows represent the position of optical source and detector. Colourbar represents the distribution between the maximum and minimum number of scattering events $\left(N_{SC}\right)$. The number of scattering distributions at different depths of tissue are shown in (**c**).

Comparatively, the distribution of scattering events through the finger tissue volume at $SaO_{2}$ = 90% in a transmittance geometry (similar to transmittance pulse oximetry, the most commonly used PPG system in clinical practice) is shown in Figure 4. The maximum numbers of scattering events occurred near the source and the detector, which were limited to dermal sublayers. A high number of scattering events were also observed in the bone part of the finger. In Figure 4c, the frequency of the scattering events are plotted, and the number of scattering events in red was found to be much higher compared to infrared. The profile of the plot stated that the bone part caused the maximum scattering, and then the upper and lower dermal sublayers. The least scattering parts were the upper and lower fat layers. In both simulations (Figure 3 and Figure 4), the detected number of photon packets were 1 × 10^8^. The average time taken for each simulation was 2 hours.

**Figure 5:**
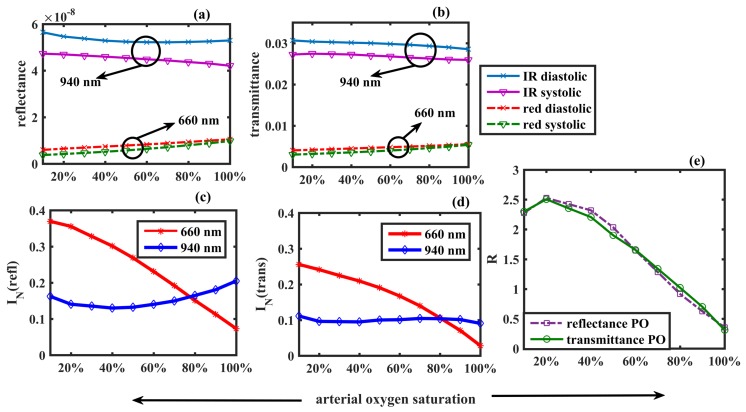
Detected reflectance and transmittance in two sets of PPG geometries are shown in (**a**,**b**). The normalised reflectance and the normalised transmittance, as functions of arterial blood oxygen saturation, are plotted in (**c**,**d**). The ‘ratio of ratios’ R is plotted against the arterial blood oxygen saturation in (**e**).

In Figure 5a,b, the detected ‘intensities’ in two PPG modalities, recorded in the same geometry as in Figure 3 and Figure 4 respectively, are presented. The overall transmittance was higher than the overall reflectance for both red and infrared wavelengths at systole and diastole. For example, at 90% oxygen saturation, the IR diastolic transmittance was 2.89 × 10^−2^, which was about 10^5^ times higher than the IR diastolic reflectance, i.e., 5.27 × 10^−8^. Apparently, both the reflectance and transmittance for red light increased very slowly with arterial oxygen saturation, whereas for IR it gradually decreased. The diastolic intensities were slightly higher than the systolic intensities in all cases. The calculated normalised pulsatile reflectance ($I_{N}\left(refl\right)$) and transmittance ($I_{N}\left(trans\right)$) are shown in Figure 5c,d respectively. Although the values of the individual detected intensities varied greatly between the two modalities, the normalised pulsatile intensities were almost identical in both cases. For example, at an oxygen saturation value of 90%, the normalised reflectance $I_{N}\left(refl\right)=0.18$ was very close to the normalised transmittance $I_{N}\left(trans\right)=0.10$. The red normalised intensity was higher than the infrared normalised intensity in both modes. The ratio of ratio (R) for two PPG modalities is plotted as a function of the arterial oxygen saturation in Figure 5e. To compute the correspondence between the ratio of ratios of the two modalities, the Pearson Product-Moment Correlation Coefficient *r* was calculated which returned the value as r=0.996, showing a very strong positive correlation.

**Figure 6:**
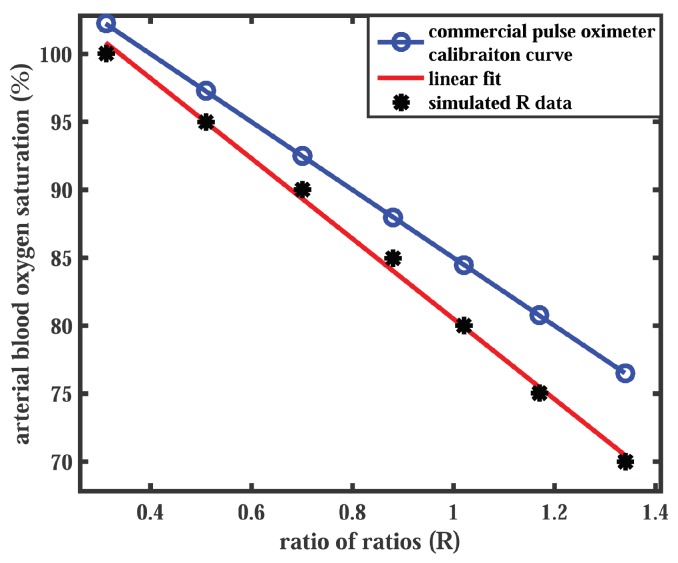
A comparison of the Monte Carlo predicted calibration curve with the commercial pulse oximeter calibration curve is presented. The simulated data points (black markers) are linearly fitted (red solid line). The commercial pulse oximeter calibration curve (blue solid line) is generated by Equation (11).

The calibration curve simulated for the transmittance mode PPG system was compared with the empirical calibration curve of a commercial pulse oximeter for reference. The relationship between the oxygen saturation measured by the commercial pulse oximeter ($SpO_{2}$) and the ratio of ratios *R* is presented by the equation for the standard calibration curve:

(11) $$SpO_{2}=110-25R$$

which has been extensively used in many applications of pulse oximetry [2,50,51,52,53]. In general, commercial pulse oximeters are calibrated empirically within the oxygen saturation range of 70–100%. For comparison, the simulated values of *R* corresponding to the same $SaO_{2}$ values are plotted in Figure 6. A linear fit to the simulated data points resulted in a linear relationship between *R* and $SaO_{2}$:

(12) $$SaO_{2}=110-29.5R.$$

A check for the Pearson Product-Moment Correlation Coefficient between the two curves returned the coefficient value as r=0.998, which is a very strong positive correlation between the simulated and the reference data.

**Figure 7:**
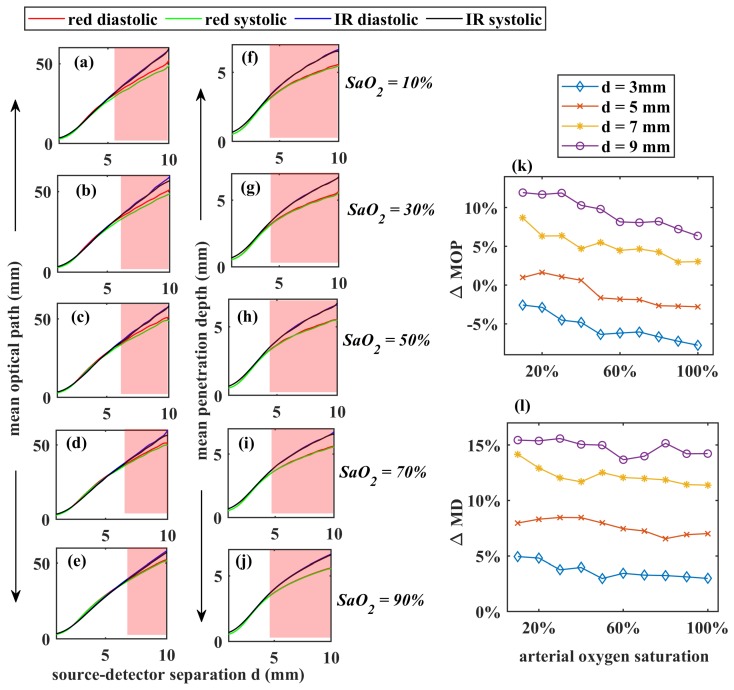
The mean optical path and the depth of penetration at red and infrared wavelengths for different $SaO_{2}$ are shown in (**a**–**j**) respectively. The percentage changes in diastolic mean optical path and penetration depth (ΔMOP and ΔMD) at d = 3 mm, 5 mm, 7 mm, and 9 mm are presented in (**k**,**l**) respectively. The shaded area in the graphs are the regions where the optical paths and penetration depths deviate between the operating wavelgnths.

For a comprehensive demonstration of the variation of the mean optical path (MOP) and the mean penetration depth (MD) of photons through finger tissue as functions of source-detector separation (*d*), the reflectance geometry was chosen. The source-detector separation was varied from 1 mm to 10 mm, with an 1 mm gap between two consecutive detections, and MOP and MD were recorded at each *d* for $SaO_{2}$ = 10%, 30%, 50%, 70%, 90%, which are shown in Figure 7a–j respectively. The total number of detected photon packets through the range of *d* were 10^10^. The simulations were performed at both the red and infrared wavelengths at systolic and diastolic states. MOP increased almost linearly with *d*. The maximum optical path taken by any photon packet was 60 mm. Penetration depth increased sharply with an initial increase of *d*, and for higher separations it increased slowly.

Interestingly, no photon passed through the finger beyond the depth of 8 mm, even for a high source-detector separation of d=10 mm. No significant difference was visible in the diastolic and systolic optical path and penetration depths. However, wavelength-dependent deviation in optical paths and penetration depths were noticed, especially at higher source-detector separations (d>6 mm) as shown in the shaded regions of the Figure 7a–j. This wavelength dependency of MOP and MD were quantified as the percentage change between their values at red and infrared (ΔMOP and ΔMD, respectively) as stated in the following the equations:

(13) $$\begin{array}{c} \Delta MOP=\frac{MOP(IR)-MOP(red)}{MOP(IR)}\times 100\\ \Delta MD=\frac{MD(IR)-MD(red)}{MD(IR)}\times 100 \end{array}$$

Calculated (ΔMOP and ΔMD) at d= 3 mm, 5 mm, 7 mm and 9 mm for $SaO_{2}$ = 10–100% are shown in Figure 7k,l, respectively. In Figure 7k, the negative values of ΔMOP at d=3 mm referred to the higher optical path at infrared compared to the red wavelength. With increasing *d*, the infrared optical path became higher than the red optical pathlength. With increasing oxygen saturation, the difference between red and infrared optical paths slowly decreased. In Figure 7l, all positive values of ΔMD indicated that penetration depth in infrared light was always higher than red. The difference in penetration depth between red and infrared light did not exhibit any significant variation with increasing oxygen saturation.

**Figure 8:**
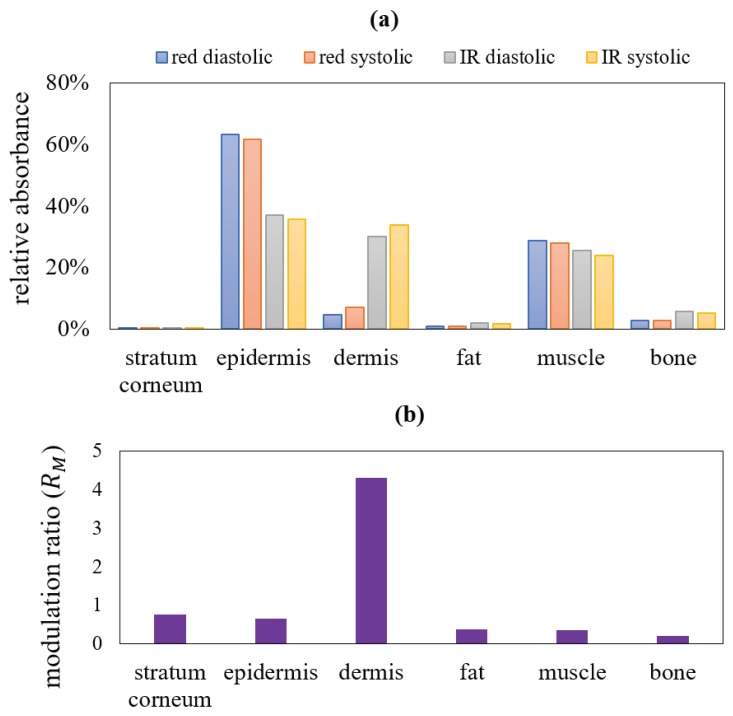
In a transmittance mode PPG, the distribution of the relative absorbances (in percentage form) and the absorbance modulation ratio $R_{M}$ at different layers are shown in (**a**,**b**) respectively. The systolic and diastolic absorbances in both red and infrared wavelengths at different tissue layers and the corresponding modulation ratio, shown in this figure, are illustrated in Table 3.

The model was further explored to retrieve the information on the absorption from each layer of the tissue. While travelling from source to detector in the reflectance geometry, light might not pass through all layers of the finger, as already seen in earlier results. To acquire the signature of all tissue layers in the total absorbance, this study was performed on the transmittance geometry only. The distribution of relative absorbances (i.e., the absorbance by each layer relative to the total absorbance by the entire tissue) is illustrated in Figure 8a. The absorbance modulation ratio $R_{M}$ is presented in Figure 8b. The absorbances are quantified in Table 3.

The maximum absorbed photons of both red and infrared wavelengths at systole and diastole were localised in the epidermis. The minimal absorption was found in the stratum corneum, then in fat, bone, muscle and dermis. Interestingly, unlike any other layer, in dermal layer the difference between the infrared and red absorbances was very high in both diastole and systole (about 7 and 5 times respectively, as shown in Table 3). Again, the tabulated data showed a higher relative systolic absorbance compared to diastolic in the pulsatile dermis, whereas the reverse was found in other non-pulsatile layers. The systolic increase in dermal blood led to not only an increase in absorption by the dermis, but also an overall increase in absorption by the entire tissue volume. Thus, the relative systolic absorption by other non-pulsatile layers (i.e., the ratio between the absorption by the layer and the total absorption by entire finger) decreased in systole from diastole. In the outermost layer, i.e., the stratum corneum, because of a very small thickness and absence of any absorber (e.g., blood, water or melanin), the absorbance is very low in both systole and diastole, and the difference between the two states are negligible (upto two decimal points, the values appear to be the same).

**Figure 9:**
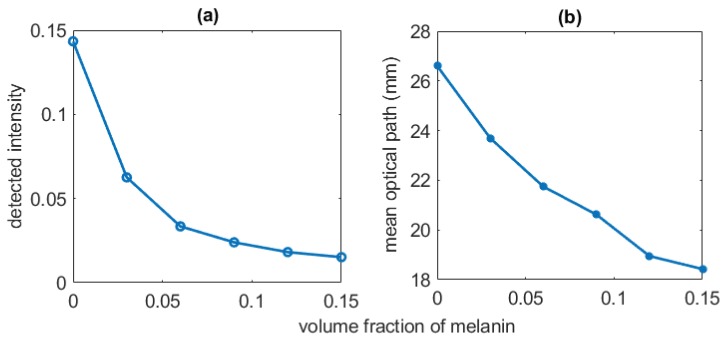
The effect of melanin concentration on the intensity (detected photon weight) and the mean optical path are shown in (**a**,**b**) respectively.

To estimate the influence of the non-pulsatile tissue components of the light-tissue interactions, the absorbance was recorded for varying fat-layer thickness and melanin concentration. In Figure 9, the effect of melanin on the variables such as detected weight (intensity) and optical pathlength is demonstrated. The melanin volume fraction was varied between 0% (i.e, skin with no melanin) to 15% (i.e., a highly pigmented skin). With increasing melanin concentration, a consistent decrease in both detected weight and optical path were apparent. In Table 4, the influence of the varying subcutaneous fat layer on the detected intensity is illustrated. Very slow decrease in the intensity (i.e., detected photon weight) with increase of fat thickness was visible at both wavelengths.

## 4. Discussion

A robust opto-anatomical model for light-tissue interaction in Photoplehysmography in a specific ROI has been presented in this paper. Monte Carlo method was chosen for simulation which is a flexible and reliable approach for computing the optical interaction with complex medium such as biological tissue. The accuracy of the method can be quantified by its convergence rate, given by $1/\sqrt{Q}$ where *Q* is the number of simulations [54]. The minimum number of simulated photon packet in this work was $Q=10^{8}$, resulting in a convergence rate of 0.0001. A high number of iterations produced a reliable and accurate result in this work. To minimise the processing time, the variance reduction technique was adapted in the algorithm and a multi-threaded computational platform was used.

It is needless to say that the biological tissue is a highly heterogeneous and complex structure, with the variable spatial distribution of blood and other chromophores in different depths. The volume distribution of blood in tissue layers, and the thicknesses of the sublayers also can vary from subject to subject. Nevertheless, efforts were made in this work to choose the parameters rationally and carefully to model an average healthy human index finger anatomy using the optical parameters based on an intense literature survey. The index finger was chosen as it is the most commonly used location for PPG sensor (i.e., pulse oximeter) in clinical setting for continuous monitoring of arterial blood oxygen saturation. In the computational experiment, the subject was considered at rest, and free from any external influences such as motion, electromagnetic interference etc. The effect of the physiological parameters of the pulsatile components such as blood volume and oxygen saturation, and those of the non-pulsatile components such as tissue thickness or melanin concentration have been separately studied in this paper for detailed understanding of the realistic model.

PPG is usually used for assessing the peripheral oxygenation and perfusion, rather than deep tissue monitoring, thus shorter source-detector separations (<1 cm) were simulated in the work. An important observation from the reflectance PPG simulation was the wavelength-dependence of the optical path and penetration depth for higher source-detector separations. In the dual wavelength PPG-applications such as pulse oximetry, it is usually assumed that the operating wavelengths interrogate the same sampling volume [15]. However, present simulation showed a considerable deviation between the red and infrared optical paths and penetration depths at higher source-detector separations. The percentage changes in the mean optical path and mean penetration depth were very low (ΔMOP ≈ 0% and ΔMD ≈ 7%) at d=5 mm, whereas those were the maximum (ΔMOP ≈ 10% and ΔMD ≈ 15%) at d=9 mm. Starting from Beer-Lambert law, the relationship between the arterial oxygen saturation and the ratio of ratios for a dual wavelength PPG system such as pulse oximeter can be derived as follows [55]:

(14) $$\begin{array}{c} SaO_{2}=\frac{\epsilon_{D_{red}}-R\left(MOP_{IR}/MOP_{red}\right)\epsilon_{D_{IR}}}{R\left(MOP_{IR}/MOP_{red}\right)\left(\epsilon_{O_{IR}}-\epsilon_{D_{IR}}\right)-\left(\epsilon_{O_{red}}-\epsilon_{D_{red}}\right)} \end{array}$$

where $\epsilon_{D}$ and $\epsilon_{O}$ are the extinction coefficients (i.e, the combination of scattering and absorption coefficient) of deoxy and oxyhaemoglobin respectively. An assumption that MOP(R)=MOP(IR), therefore, would cause an underestimation of the reading. Chances of an error is higher for the patients with compromised oxygen saturation as the percentage difference is higher for lower saturations. However, in practice the traditional pulse oximeters, which are used for clinical monitoring, are calibrated empirically, i.e., the calibration curve is produced by experimentally acquiring a large dataset from the healthy volunteers, artificially bringing them to lower oxygen saturation states [2]. Because of the empirical calibration, the traditional pulse oximeters do not exhibit any inaccuracy in their measurements. However, the empirical calibration method imposes an obvious ethical restriction to create very low level of oxygen saturation in the volunteers, resulting in a deficit in the data for $SaO_{2}$ < 70%. For a general PPG-based application, therefore, the concept of wavelength dependence of the optical path is crucial and an optimisation of the source-detector separation is of utmost importance in such applications. For a reflectance mode finger PPG system using 660 nm and 940 nm wavelengths, an optimised source-detector separation d≤6, estimated from the present work, can be used as a future reference.

The dissimilarities in the distribution of scattering at red and infrared wavelengths were caused by the higher scattering co-efficient at the lower optical wavelength. The full scattering profile in the transmittance geometry showed the maximum scattering events taking place in the bone layer, followed by the dermal layer. On the other hand, the relative absorbance was maximum in the dermal layer and minimal in the bone layer. Results confirmed that despite exhibiting a high scattering, the bone layer had a minimum impact on the detected PPG intensity. There has been no study so far that has simulated a tissue volume including the bone optical properties in reflectance and transmittance optical modalities, thus no information was available on the contribution of the bone layer in the light-tissue interactions in a system. The quantification of the relative absorbance and the modulation ratio of different tissue layers, therefore, would be beneficial for understanding PPG and other similar optometric sensor technologies.

The ‘pulse’ simulated in the model, i.e., the difference between systolic and diastolic intensities, was caused by the absorption by the pulsatile tissue compartment, contributing to the ac component of the PPG signal. The diastolic intensity, on the other hand, represented the absorption by the non-pulsatile tissue compartment producing the dc component of the PPG signal. The ratio of ratios plotted against arterial oxygen saturation resembled the calibration curve which is the technical characterisation of a pulse oximeter. The strong positive correlation between the calibration curves of the two modalities indicated that the sensor geometry does not significantly influence the efficiency of a pulse oximeter for $SpO_{2}$ measurement. With an increasing arterial oxygen saturation, the oxyhaemoglobin optical properties dominated over deoxyhaemoglobin, leading to a rapid decay in the red normalised intensity and a slow increment in the infrared normalised intensity. The variation in the normalised intensities resulted in sigmoidal shapes of the simulated calibration curves. The ratio of ratios at lower oxygen saturation is of a fundamental importance, however, clinical applications of a pulse oximeter are primarily concerned with the data above 70% oxygen saturation. In the $SaO_{2}$ region between 70% and 100%, a linear variation in R-values was found. The excellent correlation between the simulated plot and the empirical calibration curve of the commercial pulse oximeter further served as the validation of the model.

The non-pulsatile parameters such as tissue thickness and skin pigmentation often raise questions regarding the PPG measurement. The influence of these parameters were assessed using the present model. For a person with a very light-couloured skin, the detected light intensity was seen to be much higher (3–5 times) compared to a person with very dark coloured skin because of the obvious reason that the epidermal melanin is a very strong absorber of light. The decay of intensity exponentially fell with the increasing melanin concentration. The optical pathlength in tissue also was found to decrease with the increasing absorbance by melanin even though no change in scattering took place, indicating the dependence of the optical path on the absorption property of the tissue. However, no change in penetration depth was found with the increasing melanin which establishes the fact that penetration depth does not depend on the absorption coefficient of the tissue. Due to the changes in the optical pathlength, the photons will be likely to interrogate slightly different sample volumes with increased melanin concentration, resulting in an increase in noise in the measurement. In the experiment with the increasing thickness of the fat layer in the finger, no significant change in the detected light intensity was visible. Therefore, the relativistic measurement should not be affected for different finger thicknesses in a transmittance PPG setting such as pulse oximetry.

Previous research works in this field primarily focussed on the optical interactions with the cutaneous tissue structure only which is responsible for the pulsatile signal (ac component). Recent advancements in PPG research have explored the dc part of the PPG signal for potential applications [3,7,56]. To analyse the origin of the dc PPG, the contributions of *all* non-pulsatile tissue compartments had to be considered which was never addressed in earlier studies. To the authors’ knowledge, the computational model presented in this paper is the first attempt to investigate the optical interaction with all pulsatile and non-pulsatile tissue compartments of the finger, providing an insight into the formation of both dc and ac PPG signal. The results from both the reflectance and transmittance geometrical settings exhibited a localisation of photons in the dermal tissue because of the vascular network present in this layer. Dermal vasculature, which consists of capillaries, arterioles and venules, originates from the deep blood net dermis adjacent to the subcutaneous fat and extends up to the papillary dermis. It is well-established that the pulsatility is observed only in arterioles [57], which was simulated in this model by the increment in the arterial blood volume in the dermis. The volumetric increment of the arterial blood was associated with a proportional decrement in the volume of rest of the dermal tissue, i.e., the non-pulsatile tissue-compartments. This consideration manifests the physiological hypothesis that the systolic increase in blood volume in the arterioles increases the transmural pressure, resulting in a compression in the elastic fibres in the dermal connective tissue and the overall density of the capillaries present in the dermal sublayers [21].

A basic model of finger PPG has been presented in this paper. To solve the problems related to PPG, for example, the motion artifacts, pulse transit time (PTT), the effect of large artery etc., future modifications would be implemented in the model. To produce the pulsatile signal, the mechanical properties of the blood vessels need to be incorporated in the model. Future plan includes the variation in the blood volume in accordance with the blood pressure. In a tissue volume such as finger, i.e., in absence of a large artery, the simulated volume $V_{b}$ of arterial blood in the skin tissue can be related to the blood pressure under certain conditions [58,59,60]:

(15) $$P=b\cdot exp(nV_{b})$$

where *n* and *b* are the system-dependent constant terms. On the other hand, for any other tissue region that contains a large artery, for example, forehead or forehand, the stress-strain relationship between the pressure and the radius (*r*) of the artery can be written as:

(16) $$\Delta P=\frac{\Delta r\cdot E_{p}}{r_{0}}$$

where Δr is the change in the artery radius due to a ΔP change in the pressure, $E_{p}$ being the stress-strain modulus of the artery [61,62]. The elastic properties will be included to the tissue model using the Equations (15) and (16) which will be varied between the diastolic and systolic blood pressure, corresponding to the cardiac cycle, to produce the PPG waveform. Such a model then may be applied for investigating the existing problems related to PPG as already mentioned before.

## 5. Conclusions

The optical interaction with a three-dimensional finger tissue volume in a dual wavelength PPG system was analysed using the Monte Carlo computational tool. The model was executed at the reflectance and transmittance PPG geometry to investigate the light-tissue interaction parameters relative to different sensor specifications (i.e, wavelength and source-detector separation) and different tissue physiology (i.e., volume and oxygen saturation of arterial and venous blood) in the presence of multiple absorbers (e.g., oxyhaemoglobin, deoxyhaemoglobin, water and melanin). The results identified the optimal source-detector separation for a reflectance mode finger-PPG sensor. Depth-specific analysis of the tissue-volume revealed the maximum and minimum impacts of the dermis and bone, respectively, in the formation of a PPG signal. Results explained the origin of the pulsatile and non-pulsatile PPG components which is an invaluable information for designing a wearable finger PPG probe for continuous physiological measurements. All results presented in the paper contribute to the basic knowledge required for the development and advancement of the novel PPG-based transcutaneous sensors.

## Figures and Tables

**Table 1:** The values of the parameters used to model the dermal sublayers that were adapted from literature [13,17,23,34,36,37].

Dermal Sublayer	t (mm)	$V_{b}$	$V_{w}$
stratum corneum	0.02	0	0.05
epidermis	0.25	0	0.2
papillary dermis	0.1	0.04	0.5
upper blood net dermis	0.08	0.3	0.6
reticular dermis	0.2	0.04	0.7
deep blood net dermis	0.3	0.1	0.7

**Table 2:** Optical properties of tissue layer in finger tissue model.

Tissue Component	$\mu_{a}$ (mm^−1^)		$\mu_{s}$ (mm^−1^)		*g*
	660 nm	940 nm	660 nm	940 nm	
skin	-	-	25.62	15.68	0.9
fat	0.0104	0.0170	6.20	5.42	0.8
muscle	0.0816	0.0401	8.61	5.81	0.5
bone	0.0351	0.0457	34.45	24.70	0.92
oxyhaemoglobin	0.15	0.65	-	-	-
deoxyhaemoglobin	1.64	0.43	-	-	-
water	0.0036	0.2674	-	-	-

**Table 3:** Simulated distribution of relative absorbances and modulation ratio in tissue layers.

Tissue Layers	Red Diastolic	Red Systolic	Infrared Diastolic	Infrared Systolic	$R_{M}$
Stratum corneum	0.12%	0.12%	0.09%	0.09%	0.77
epidermis	63%	61%	37.07%	35.57%	0.66
dermis	4.53%	6.97%	29.99%	33.74%	4.30
fat	0.82%	0.79%	1.79%	1.64%	0.37
muscle	28.58%	27.89%	25.50%	23.75%	0.36
bone	2.83%	2.79%	5.56%	5.21%	0.21

**Table 4:** Relationship between detected photon weight and the thickness of fat layer.

Fat Layer Thickness (mm)	Detected Photon Weight	
	660 nm	940 nm
0.2	0.025	0.039
0.4	0.0229	0.035
0.6	0.0226	0.035
0.7	0.0220	0.032
0.8	0.0218	0.030
0.9	0.0214	0.029
